# Assessment of Stability and Degradation Kinetics of Carnosic and Rosmarinic Acid in Edible Oil and Its Effectiveness as an Extraction Medium Compared to Other Solvents

**DOI:** 10.3390/molecules30224394

**Published:** 2025-11-13

**Authors:** Agnieszka M. Hrebień-Filisińska, Sylwia Przybylska

**Affiliations:** Department of Fish, Plant and Gastronomy Technology, Faculty of Food Sciences and Fisheries, West Pomeranian University of Technology, 71-459 Szczecin, Poland; sylwia.przybylska@zut.edu.pl

**Keywords:** carnosic acid half-life, stability rosmarinic acid, degradation rate, kinetic model, ecological extraction

## Abstract

The aim of this study was to compare the effectiveness of edible oil (as an extractant) for the extraction of CA (carnosic acid), C (carnosol), and RA (rosmarinic acid) from sage with popular solvents (methanol, ethanol, 70% methanol, 70% ethanol, and water), as well as to assess the stability and fit the kinetic reaction model to the course of CA and RA degradation in oil macerate and various extracts. The degradation rate constant and half-life of CA and RA were also estimated and compared. CA was most efficiently extracted from sage using ethanol and methanol (9.3–10.1 mg/g of sage), followed by oil (7.10 mg/g). For C and RA, the most suitable solvents were 70% ethanol and 70% methanol (C: 3.08–4.01 mg/g; RA: 19.16–20.24 mg/g). CA was most stable in oil, followed by ethanol > methanol > 70% ethanol > 70% methanol. CA degradation followed a first-order kinetic model. RA was very stable in all extracts, except water, where the kinetics of RA degradation most closely followed a second-order model. Although oil extracts smaller amounts of phytochemicals from sage than solvents, CA in oil was the most stable, and the maceration of sage in oil is safe and consistent with the concept of sustainable development.

## 1. Introduction

Carnosic acid (CA) and its derivative carnosol (C) and rosmarinic acid (RA) are natural compounds of plant origin with extremely strong antioxidant properties. They are also credited with anti-inflammatory, anti-cancer, anti-aging, neuroprotective, anti-diabetic, antibacterial, and antiviral activity [1,2,3,4]. There is also evidence that CA can lower blood triglycerides, cholesterol, and glucose levels [5] and show great activity in the treatment of PIH (post-inflammatory hyperpigmentation) [6]. RA has cardioprotective, hepatoprotective, nephroprotective, antidepressant, and antiallergic effects [3]. It may also have a beneficial effect on skin health, which is why it is used in sunscreens as a photoprotective agent and to alleviate the symptoms of dermatitis [2].

CA is composed of 20 carbon atoms, 28 hydrogens, and 4 oxygens (C_20_H_28_O_4_); its structure comprises abieta-8,11,13-triene substituted by hydroxy groups at positions 11 and 12 and a carboxy group at position 20 [7]. CA contains a phenolic group and is often classified as a polyphenol, but its genesis, biosynthetic pathway, and properties allow it to be classified as a terpene (isoprenoid) [7]. RA is an ester of caffeic acid and 3,4-dihydroxyphenyllactic acid. The molecular formula of RA is C_18_H_16_O_8_; RA is a phenolic acid and acts as an effective antioxidant by trapping free radicals [8].

CA and RA are most abundant in rosemary and sage. Thanks to these compounds, these plants have a healing and therapeutic effect on the body. Therefore, they are quite commonly used to prepare various extracts, water infusions and herbal teas, which are used for various ailments. They are also widely known for their aromatic properties and are often used in the kitchen as herbal spices to improve the taste and aroma of dishes, as well as to extend the shelf life of food. Due to their high antioxidant activity, mainly CA—but also RA—is used in the production of antioxidant preparations used to inhibit oxidation in food, as well as active substances in pharmacological or cosmetic agents. RA, C, and CA are most often obtained in the process of extracting the leaves of *Rosmarinus officinalis* or *Salvia officinalis* using a system of solvents approved for contact with food, or by extraction with supercritical carbon dioxide. The selection of an appropriate solvent and extraction method for the physicochemical properties of bioactive components affects the achievement of the appropriate efficiency of the extraction process and their high concentration in the extract. Good results are obtained by using unconventional methods (e.g., supercritical fluid extraction, pressurized liquid extraction, high hydrostatic pressure extraction, pulsed electric field extraction), but they are associated with high investment costs [9]. On the other hand, conventional methods, in which organic solvents are used for the extraction of antioxidants, are very time-consuming, require relatively large amounts of solvents, which is not in line with the principles of sustainable development and is a problem for the environment [9]. Recently, “green” extractants such as polyethylene glycol (PEG) [10], bio-based solvents [2] or a type of green thermo-switchable solvent system [11] have attracted attention in research.

The final concentration of active substances in extracts may also be influenced by degradation processes. CA is particularly unstable, easily degrading in organic solvents, at elevated temperatures, and under the influence of light [12,13]. Although the breakdown products of CA still retain their strong antioxidant properties, they may become completely depleted over time. Therefore, the search for ecological methods and extractants that will not only ensure high extraction efficiency of bioactive substances from sage or rosemary, but also guarantee their high stability, is very important and timely. Therefore, the method of extracting phytochemicals using edible oils, the so-called oil maceration, seems interesting and can be classified as a “green extraction method” because it does not require the use of organic solvents and has no harmful effect on the environment and health. It is a natural, completely safe, and simple method of extracting active ingredients from plant material using edible oils. The preparations obtained in this way, rich in health-promoting phytochemicals, can be an ideal enriching ingredient for food or cosmetics, e.g., from the group of natural or ecological products. Our recent studies have shown that the use of fish oil enables the extraction of CA, C, and RA directly from sage and obtains an oil macerate rich in phytochemicals derived from sage [7,14]. We also obtained similar results in the case of linseed oil [15]. However, so far, the effectiveness of oil maceration has not been compared to standard extraction methods based on popular solvents. There is also a lack of data on the stability of CA, RA, and C in the macerate obtained by the process of sage maceration in oil, assisted by ultrasound. According to research, CA from commercially available rosemary extract, when added to fish oil, is relatively stable [13]. Similarly, in our recent research [14], CA, RA, and C, after maceration assisted by homogenization, were quite stable in oil. However, the data obtained are incomplete and one can only assume that CA in macerate, after the use of ultrasound, will also maintain a long shelf life. However, it is not fully known how ultrasound will affect the stability of phytochemicals in macerate and whether phytochemicals will not degrade during storage of macerate. There is a suspicion that with appropriate extraction parameters, intensive cavitation not only accelerates the process of polyphenol extraction from plant tissues and improves extraction efficiency, but on the other hand can initiate radicals that can initiate polyphenol degradation [16]. There is also no comparative assessment of the kinetics of the degradation rate of CA and RA in different extractants. Therefore, it was decided to investigate whether maceration of sage in oil, assisted by ultrasound, is a more effective method of extracting CA, C and RA than extraction with ethanol, methanol, their aqueous solutions (70%), and water, and to examine the stability of CA, C, and RA in the obtained oil macerate and extracts during their storage.

The aim of this study was to evaluate the effectiveness of the oil as an extractant for the extraction of CA, C, and RA from sage in comparison to typical organic solvents and water, as well as to compare the stability and kinetics of the degradation rate of CA and RA in different types of extractants.

## 2. Results and Discussion

### 2.1. Effect of Solvent Type and Extraction Method on the Extraction of Carnosic Acid (CA), Carnosol (C) and Rosmarinic Acid (RA)

Oil (O) was used for extraction by maceration (MA). For comparison, extractions were also carried out in an ultrasonic bath (U) and in a water bath (B) using the following solvents: methanol (M), 70% methanol (70M), ethanol (E), and 70% ethanol (70E). Water (W) was used to prepare sage infusions (I). The extraction efficiency of CA depended mainly on the type of solvent (Table 1). CA was most efficiently extracted using ethanol (E) and methanol (M), reaching an average content of 9.3–10.1 mg per g of sage. There were no significant differences between ethanol and methanol (although ethanol seemed to be slightly more efficient), nor between “cold” extraction (U) carried out in an ultrasonic bath (temp. 20–25 °C) and “hot” extraction (B) in a water bath (decoction). The next in the ranking in terms of CA extraction efficiency was oil (MA/O′), at a similar level with 70% ethanol and 70% methanol. The content of CA in the samples: MA/O′, B/70M, B/70E, U70/M, U/70E, was comparable, reaching a value from 6.92 to 7.54 mg/g of sage. In the case of oil macerate, during analyses to determine the active ingredients, they were extracted from the oil using methanol and methanol 70%. Methanol extracts from oil (MA/O′) contained more CA than extracts based on 70% methanol (MA/O″), similarly to sage extracts. The lower content of CA in extracts based on 70% methanol may be related not only to the extraction efficiency, but also to the low stability of CA in water–alcohol solvents. It should be emphasized that in the case of themacerat analysis, a methanol or methanol–water extract was injected into the chromatograph column, thus processing the macerate in some way to determine the compounds. However, the remaining extracts were injected directly into the column. Therefore, it can be assumed that some of the CA, although extracted from the sage into the oil, was not fully extracted to M or 70M, so the actual CA, C, or RA content may be even higher. However, in the case of direct solvent extraction of the sage, there is also no guarantee that all components were recovered.

The extraction efficiency of 70% ethanol with respect to CA was similar to 70% methanol and significantly lower than ethanol or methanol. However, CA could not be extracted using water, because this phytochemical was not determined in sage water teas (I/W).

It was a bit different in the case of carnosol (C). The highest concentration of C (3.08–4.01 mg/g) was determined in methanol–water and ethanol–water extracts, regardless of the extraction method (B/70M, U/70M, B/70E, U/70E). Lower C content was in methanol (2.2–2.31 mg/g) and oil (1.03 mg/g). In oil, the C level depended on the solvent used to identify CA, a much higher C concentration was determined when 70M (2.15 mg/g) was used for analysis than M (1.03 mg/g). However, in ethanol extracts (B/E, U/E) after the extraction process, carnosol was not identified. It was similar in the case of water infusions of sage (W/I), in which carnosol was also not identified.

RA was best extracted when 70% ethanol and 70% methanol were used for the extraction process, in samples B/70M, B/70E, U70/M, U/70E; the concentration of RA was the highest, at the level of 19.16–20.24 mg/g. Lower RA contents (11.5–12.45 mg/g) were determined in aqueous extracts of sage, obtained by infusion (I/W) and in methanol extracts obtained using a water bath (B/M). Even lower RA concentrations (7.56–8.10 mg/g) were found in methanol extracts, after sonication (U/M) and in ethanol extracts obtained “hot”, in a water bath (B/E). The U/E method was also less effective for RA extraction (3.86 mg/g). The lowest RA concentration was determined in oil macerates (0.11 mg/g)—when 70% methanol (MA/O″) was used for the determination—whereas when methanol was used for the analysis, RA was not determined at all. It was also noted that when ethanol and methanol were used for RA extraction, its extraction efficiency was dependent on the extraction method. Extraction was more effective when conducted at elevated temperatures in a water bath (B), compared to an ultrasonic scrubber (U). However, the use of aqueous solutions of both alcohols (70M and 70E), which were more optimal, resulted in comparable RA extraction efficiency, regardless of the extraction method (U or B), and therefore also the temperature. Therefore, it can be concluded that for less optimal solvents in relation to RA, better extraction efficiency can be achieved at higher extraction temperatures. However, in the case of a more optimal solvent, the extraction method has nosignificant effect.

The obtained results are similar to those in other works [17,18]. As the concentration of ethanol in the mixture with water increases (from 30 to 100%), the extraction efficiency of CA usually increases, and the extraction efficiency of RA decreases, with the extraction of RA drastically decreasing after exceeding the concentration of about 75–80%, while it is most effective with 30% ethanol. In the study of Martins et al. [19], methanol/water extract (80%) of sage contained more RA (93.22 ± 0.12 mg/g dried extract) than the aqueous extract of sage obtained by infusion (73.97 ± 0.15 mg/g dried extract), with the highest concentration detected in the aqueous extracts obtained by decoction (93.46 ± 0.64 mg/g dried extract). Similarly, in another study [20], RA was successfully extracted from Lemon Balm (*Melissa officinalis* L.) by supercritical water extraction.

The best solvent for CA, among various ethanol–water mixtures, is most often concentrated ethanol [2,17,18]. Similarly, in the case of methanol, also in other studies [21], its effectiveness in CA extraction is confirmed. However, water fails to extract CA from sage [21]. In the case of oil, its use for CA extraction seems very interesting and unconventional. In our previous experiments, CA was also successfully extracted directly from sage into fish oil during maceration assisted by homogenization or ultrasonic extraction [7,14]. To our knowledge, apart from the above studies, no research has been published to date that would indicate oil maceration as an effective method of extracting CA directly from sage into edible oil. However, for the first time the extraction efficiency of oil is compared with several standard solvents simultaneously: ethanol, methanol, 70% ethanol, and 70% methanol. As the results show, although the use of oil does not give the best results, it allows us to obtain similar extraction efficiency of CA from sage as 70% ethanol, 70% methanol. Moreover, the extraction of active ingredients using oil is a simple and safe method, friendly to the environment. In summary, the best substances for CA extraction were ethanol and methanol, followed by oil, tied with 70% ethanol and 70% methanol, while water (I/W) was found to be ineffective.

The extraction efficiency and concentration of phytochemicals in extracts are greatly influenced by their chemical structure and solubility in solvents used for extraction [17]. CA is considered a more lipophilic than polar component [7], which is mainly due to its hydrocarbon skeleton, which gives it a relatively nonpolar character [22]. On the other hand, its molecule also has functional groups: carboxyl and phenolic (hydroxyl), which to some extent give it slightly polar properties [22]. Therefore, CA dissolves better in less polar solvents (ethanol, methanol) than in more polar ones (70% ethanol, 70% methanol) and is better extracted with them. Therefore, water did not extract CA, while the use of oil enabled its extraction. In teas (I/W), RA was identified, which has a greater affinity for water than for oils. Water is characterized by greater polar properties than methanol and methanol is greater than ethanol [23]. Solvents, according to decreasing polarity, can be arranged as follows: water ˃ 70% methanol ˃ 70% ethanol ˃ methanol ˃ ethanol.

In general, RA is a component with polar properties (containing, among others, five hydroxyl groups), while at the same time it is characterized by limited solubility in water, which results from the relatively nonpolar aromatic ring structure. Hence, the extraction process and obtaining of this compound is usually carried out using organic solvents of medium polarity [24].

In the case of oil, its strong nonpolar properties determined the extraction of CA, but pure ethanol and methanol were better in this respect, which results from their physicochemical properties. Moreover, as reported by Jacotet-Navarro et al. [17], although the solubility of the active compounds determined in the solvents used for extraction has a large impact on the efficiency of this process, it is not the only factor. Extraction is a sequential molecular process in which three stages can be distinguished: 1. diffusion of the solvent through the core of the plant material, 2. desorption of the target compound from the plant matrix due to chemical affinity for the solvent, and 3. mass transfer of the solute from the vicinity of the plant to the solvent [17]. Analyzing this extraction process, one can imagine that oil is much more difficult to diffuse and penetrate into the plant material than alcohol or water due to, among other things, its larger molecules (long chains of fatty acids). All the more so because most polyphenols accumulate mainly in the vacuole of plant cells [17], the passage of oil through cellular structures to get to the vacuole may be particularly difficult.

The concentration of C in various systems is also noteworthy, and is not at all obvious. C, like CA, is considered a more lipophilic than polar component, but when analyzing the retention time in HPLC analysis (time of leaving a column with hydrophobic packing), it can be assumed that its lipophilic properties are slightly lower than CA, but definitely higher than RA [7]. However, its highest concentration was determined not in less polar solvents (ethanol, methanol) or in oil, but in methanol–water and ethanol–water extracts, while it was not identified at all in ethanol extracts. Could it be that ethanol is more nonpolar than C and the solubility of C in ethanol was impaired? Certainly not, because, for example, rosmarinic acid, which is one of the most polar compounds of sage, was extracted using ethanol. Most likely, the absence of C in ethanol extracts may be related to the relatively high stability of CA in ethanol, compared to other solvents, as C is formed as a result of CA degradation (more space is devoted to this issue in the next subsection).

The extraction efficiency is also influenced by the ratio of solvent to sage mass [17]. It should be emphasized that the sage-to-extractant ratio was different for the oil macerate than for solvent extraction. The sage-to-oil ratio in the macerate was 1:5.7 (*w*/*w*), as previous studies have confirmed that this ratio allows for obtaining the most concentrated sage oil macerate in bioactive components without additional processes (concentrating, preconcentrating). However, during the extraction of sage components using solvents, 0.05 g of sage per 50 mL of solvent was used, which is approximately 175 times higher than in the case of oil macerate. Therefore, it is very likely that the solvent-to-sage ratio used did not limit the extraction of bioactive components from sage. A higher solvent-to-material mass ratio generally allows for higher extraction efficiency [17]. This ensures that the extraction efficiency using solvents was not reduced in any way compared to oil maceration, although we wanted to emphasize the high extraction capacity of the oil. A higher oil-to-sage mass ratio could likely result in a generally higher extraction efficiency per gram of sage, but this is not certain, as extraction may be limited by the solubility of bioactive compounds [17].

### 2.2. Stability of Carnosic Acid (CA), Carnosol (C) and Rosmarinic Acid (RA) in Oil and in Various Solvents

**Figure 1:**
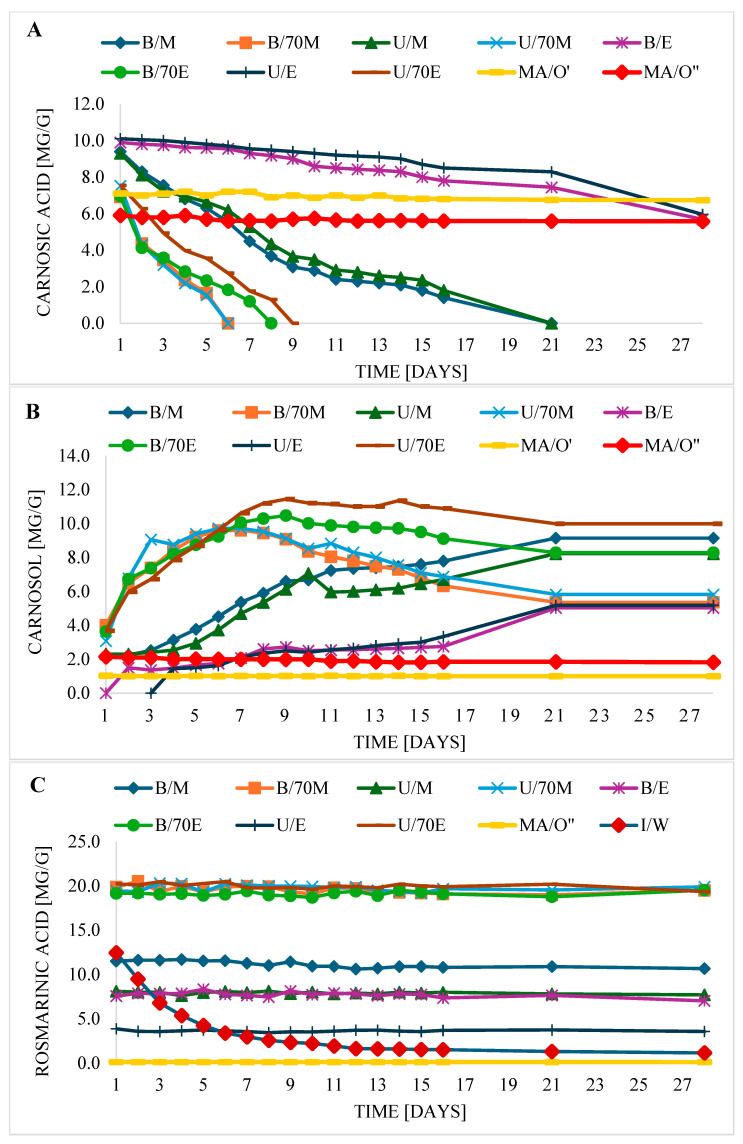
Changes in the concentration of carnosic acid (**A**), carnosol (**B**), and rosmarinic acid (**C**) in the oil macerate and in various extracts during 28-day storage (W—water, 70M—70% methanol, M—methanol, 70E—70% ethanol, E—ethanol, O—oil; extraction method: U—in an ultrasonic bath, B—in a water bath, I—infusions, MA—maceration; ′ methanol was used for the analyses; ″ 70% methanol was used for the analyses).

CA stability depended mainly on the type of solvent and less on the extraction method (Figure 1A). CA was the most stable in oil macerate (MA/O′); during 28 days of storage, no significant changes in its concentration were observed. Therefore, monitoring of CA, C, and RA retention in oil macerate was extended to 15 weeks. As shown by the data (Table S1), the CA level in the oil was unchanged for 6 weeks; only in week 15 (MA/O′ extract), a slight decrease (about 8.6%) of its amount was observed, compared to the initial day (day 1). It can therefore be concluded that ultrasound used during maceration did not significantly affect the stability of CA during storage. It was feared that ultrasound-induced cavitation could not only facilitate the extraction of polyphenols from plant tissues during maceration, but could also initiate polyphenol degradation [16]. Similarly, in our previous studies, we observed high CA stability in the macerate obtained by maceration of sage in fish oil, aided by homogenization [14], but to determine its level during analyses, 70% methanol was used, which is more universal for all polyphenols, but may be suboptimal for CA. Subsequent studies have shown that methanol is better for this type of identification [7], hence in this study, concentrated methanol was used to determine CA in oil macerate, in addition to 70%. In the study by Abedi et al. [25] after 14 days of storage of sausage with sage extract, a reduction of about 5% in CA was found and about 13% in C, compared to the initial day. Therefore, the stability of CA was lower than in our study in oil, which may be due to the presence of water in the sausage. The high stability of CA in fish oil after dissolving rosemary extract in it was also confirmed in the study by Zhang et al. [13]. The same authors found it to have much higher stability in oil than in ethanol. Similarly to our study, after 28 days, the CA content in ethanol extracts decreased, depending on the extraction method: by 41% for U/E and by 43.4% for B/E, while in oil the CA level practically did not change. In turn, the stability of CA in ethanol was greater than in methanol. In methanol extracts after 21 days (U/M, B/M), CA was practically gone, which most likely indicates its decomposition. Therefore, CA degradation occurred much slower in ethanol than in methanol. So far, no studies have compared the stability of CA in these two most popular solvents. It is known, however, that CA in methanol solution is unstable; in the study by Liu et al. [12], after 32 h of storage at room temperature, it was completely decomposed. It has already been noted earlier that CA is also more stable in nonpolar organic solvents (e.g., DMSO), and less in polar ones, such as methanol [26]. Ethanol is definitely less polar than methanol, and methanol less than water [26]. The stability of CA in this study was similar to the polarity of solvents. Similarly, the oil is very nonpolar, and the stability of CA in it was the highest (Figure 1A). CA was characterized by lower stability (than in methanol) in 70% ethanol, and the lowest in 70% methanol, which may be due to the very high sensitivity of CA to the presence of water [27]. Moreover, with the addition of water to the solvent, its polarity increases, which would confirm the statement that the lower the polar properties of the matrix for CA, the greater its stability. According to Razboršek & Ivanović [26], CA degradation also occurs to a greater extent in a protic solvent (ethanol) than in an aprotic solvent (tetrahydrofuran). In the case of water–ethanol extracts, faster degradation of CA was demonstrated in B/70E extracts than in U/70E, which in turn may be related to the effect of high temperature during extraction. A similar trend was also observed for methanol (B/M and U/M) and ethanol (B/E and U/E) extracts. According to Zhang et al. [13], CA is an unstable component, and with increasing temperature and exposure to light, CA degradation increases.

In general, during storage of the extracts, there was a linear decrease in CA content with time. After 2–3 days, a 50% decrease in CA was observed in the 70M extracts (B/M, U/M). Similarly, in the B/70E and U/70E extracts, after 3–4 days, the CA content was halved compared to the initial day. In the case of B/E and U/E, however, after 28 days, CA losses were approximately 43%. In the macerate (MA/O′) after 15 weeks, only approximately 8.6% loss was observed compared to the initial day.

The C content is strongly related to the stability of CA and both compounds always occur in combination with each other, as C is formed as a result of oxidation and ring closure of CA [26].

When looking at the stability graphs of C and CA (Figure 1A,B), it can be seen that where the fastest degradation of CA was observed, i.e., in alcohol–water extracts (B/70M, B/70E, U/70M, U/70E), the C concentration increased the fastest. From the beginning of storage in these samples (B/70M, B/70E, U/70M, U/70E) until about day 6–9, the C content increased steadily, while the CA content decreased until the complete degradation of CA, which occurred on day 6–9 (Figure 1A,B). Then, after the stage (increase in C concentration), its decrease was observed. Analogous results were observed in methanol and ethanol extracts (B/M, B/E, U/M, U/E), with the increase in C occurring much slower in ethanol extracts than in methanol extracts, until stabilization. Probably after 28 days, in the next stage, the C content would decrease with time. CA is easily degraded and as a result of these reactions, CA derivatives are formed, including C. However, over time, CA and its degradation products may completely decompose. According to Liu et al. [12], CA can first decompose into carnosic acid quinone, which can be rebuilt back to CA or can be further reduced to C. Carnosol in turn, can further decompose, forming epirosmanol, rosmanol, 7-methoxyrosmanol, and 7-methoxy-epirosmanol (the end products of carnosic acid degradation in methanol). In the studies by Zhang et al. [13], significantly slower degradation of C was observed in the solution of the mixture with carnosic acid and rosmarinic acid in ethanol than when C was present alone, which may be due, as the authors explain, to the protective effect of other antioxidants present in the mixture or to the compensatory conversion of CA to C. In the case of macerates (MA/O′, MA/O″), the stability of CA and the level of C were practically unchanged over the monitored period. It is worth noting that although C could not be extracted directly from sage using ethanol (and at the beginning of storage, it was not determined at all in ethanol extracts), on the 2nd day of storage, it was identified in B/E, and on the 4th day—in U/E. Then, after this time, its level in B/E and U/E increased successively (Figure 1B). It can therefore be assumed that C was initially not present in sage tissues, which is why it was not identified in ethanol extracts. However, over time, most quickly in 70% methanol and 70% ethanol, then in methanol, and slowest in ethanol, C was formed from CA, and over time its concentration in extracts increased. Ethanol among the tested solvents (except for macerate) showed the highest stability for CA, hence during extraction CA did not degrade enough to produce C in the first days of the experiment, and only on the 2nd and 4th day was C identified in ethanol extracts. Therefore, it could be assumed that C may come exclusively from the decomposition of CA and may not be produced by the plant originally. How can we explain the presence of C in the oil macerate, since CA showed greater stability in it than in ethanol? Namely the method of analysis used for extraction and identification of active compounds in the macerate. Methanol and 70% methanol were used to mark phytochemicals in the macerate in order to initially separate them from the fat phase, which are more conducive to the degradation of CA than ethanol, hence the presence of C in the macerate, because during the extraction process from the macerate to the hydrophilic phase, or during separation on a CA column, it could have been partially transformed to C. The above results also allow us to conclude that the determination and identification of CA may be burdened with high measurement uncertainty related to its high instability; therefore, during HPLC analyses, the extracts should be injected into the chromatographic column immediately after preparation, as was the case in this study. Moreover, the use of water acidified with 5% acetic acid (along with acetonitrile) for HPLC elution may contribute to obtaining more reliable results, because CA is more stable in a low-pH environment and the addition of acetic acid improves its stability [21].

RA in most extracts was stable, which is consistent with the results of other authors [13]. However it is revealing that RA is not stable in water. With storage time, the level of RA in sage teas (W/I) decreased gradually (Figure 1C). At the beginning, its decline was very rapid. After about 3–4 days, its content decreased by about 50%. However, after a week, an approximately 76% decrease in its content was observed. No studies have been found on the persistence of RA in water, this is probably the first such study. Until now RA was considered quite stable. In the study by Zhang et al. [13], RA both when present alone in ethanol solution and in a mixture with CA and C did not undergo significant degradation under any conditions during the 13-day study. However, when in water in our study after 13 days, its concentration decreased by about 87%. It is worth adding that RA in sage extracts occurred without the presence of CA or C. Can we therefore assume that the absence of these antioxidants could have influenced its strong degradation? Probably yes, it is also possible, analogously to the stability of CA, to suppose that the stability of RA may be related to the polarity of the solvent it is in. While RA is stable in less polar solvents (methanol, ethanol, 70% ethanol, 70% methanol, oil), in water, which is more polar than aqueous solutions of alcohols, RA is not stable.

### 2.3. Degradation Kinetics of Carnosic (CA) and Rosmarinic Acids (RA)

In order to match the results to the appropriate reaction model, the graphs of the dependence of the concentration of CA, C, and RA on time were analyzed. The graphically presented data excluded that the reactions proceeded in accordance with the zero-order model (there was no linear decrease in concentration), therefore the first- and second-order kinetic models were taken into account for further analyses. However, in the case of C, the data did not fit any of the models, because in the alcohol and alcohol–water extracts, an increase and then a decrease in the level of C was observed first. In the case of RA, the kinetic model was analyzed only in one extract: the aqueous one (I/W), because in the other extracts, no changes in concentration were observed during the tested time. Previous studies have noted that polyphenol degradation usually follows a first-order kinetic model [25,28,29]. However, to increase certainty, the data were also analyzed using a second-order model. For this purpose, graphs were drawn: from the dependence of the logarithm of the concentration of a given compound (lnC) on the storage time t (for the first-order model) and the reciprocal of the concentration (1/C) on time t (for the second-order model), the value of the fit index R^2^ was determined, which was used to indicate the appropriate model, and then the (algebraic) reaction rate constant k and half-life T_1/2_ were calculated based on the formulas (Table 2). The analysis of the R^2^ values for CA showed that the degradation proceeds according to the first-order reaction model (Table S2); only in the case of RA degradation in the water extract (I/W), the reaction followed the second-order model rather than the first-order model (Table S2).

**Figure 2:**
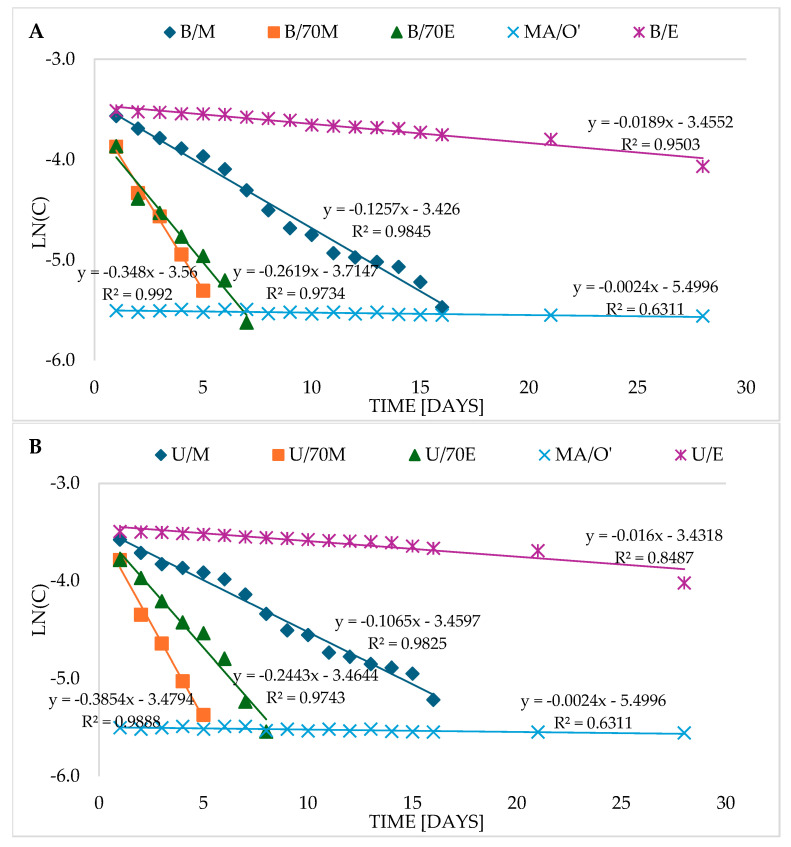
Dependence of the logarithm of the concentration (lnC) of carnosic acid on the storage time in the macerate (MA/O′) and in the extracts obtained using a water bath—B (**A**) and ultrasound—U (**B**); 70M—70% methanol, M—methanol, 70E—70% ethanol, E—ethanol, O—oil; extraction method: U—in an ultrasonic bath, B—in a water bath, MA—maceration; ′ methanol was used for the analyses.

**Figure 3:**
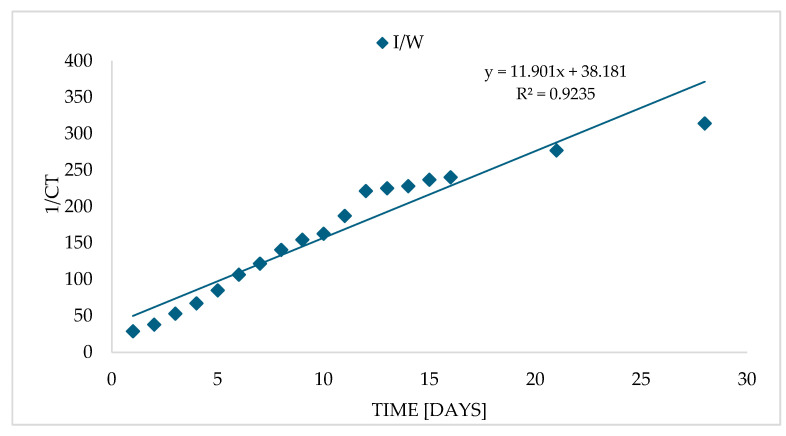
Dependence of the reciprocal concentration (1/C) of rosmarinic acid on storage time in aqueous extract (I/W).

CA in the sage oil macerate was very stable (Table 2, Figure 2A,B) and therefore both in the case of the first-order reaction (R^2^ = 0.6311) and the second-order reaction (R^2^ = 0.6375) the R^2^ coefficients determined for the macerate indicated a rather poor fit (Table S2). However, further simulations of the reaction course, in accordance with the literature, were conducted according to the principles of the first-order reaction, which seemed more appropriate. In the remaining extracts, the fit coefficient R^2^ indicates a very good fit of the CA degradation reaction course to the first-order model (R^2^ from 0.9503 to 0.9929); only in the case of the U/E ethanol extract, the fit (R^2^) is slightly lower (R^2^ = 0.8487) (Table S2). Similarly in the study by Abedi et al. [25], the first-order kinetic model fitted well to the course of CA degradation during 14-day storage of sausage with 1% sage extract. The CA degradation reaction in the macerate (MA/O′), as already mentioned, was very slow, and the calculated reaction rate constant k (k = 0.00188) was the lowest compared to the other extracts, while the half-life T_1/2_ was the highest (Table 2). The ln(C) graph for CA in the macerate (MA/O′), compared to both extracts obtained in a water bath (B) (Figure 2A) and by ultrasound (U) (Figure 2B), is actually a horizontal line with the smallest angle of inclination relative to the X-axis (slope = 0.0024), which indicates a very low reaction rate. It therefore seems that the slope coefficient can approximately indicate the reaction dynamics as a graphical determinant of the reaction rate k. The smaller it is and the smaller the slope of the graph relative to the X-axis, the slower the reaction rate, and vice versa. In this case, the value of the slope coefficient (0.0024) was on average comparable to the algebraic rate constant k, determined from the formula (k = 0.00188), and the half-life T_1/2_ calculated on its basis differs quite significantly from the calculated (algebraic) half-life T_1/2_. Nevertheless, the trend of changes in both methods of analyzing the reaction kinetics are quite consistent with each other. Hence, it can be concluded that the data obtained on the basis of the function graphs (Figure 2 and Figure 3) can be helpful in interpreting the results, but the analysis should be based on the results obtained from the proper calculations.

In alcoholic and alcohol–water extracts B and U, CA degradation occurred much faster than in macerate. Table 2 presents a ranking of extracts from the most stable for CA to the least stable, arranged based on the value of the rate constant k and the half-life T_1/2_. The results indicate that after maceration, the most stable for CA, among the alcoholic extracts, were ethanol extracts. As indicated by algebraic data (Table 2), the CA degradation reaction in the extract (B/E) was about ten times faster than for macerate, and the half-life T_1/2_ was about ten times shorter (Table 2). The CA decomposition reaction occurred at a similar but slightly higher rate in U/E. CA decomposed about 5 to 6 times faster than in U/E in methanol extracts (B/M and U/M) and its half-life was about 5–6 times shorter. The CA degradation reaction was even faster in ethanol–water extracts (70%), and the fastest in methanol–water extracts (70%). The decomposition of CA in U/70M was about 169 times faster than in macerate, and the half-life was 1.8 days and was 169 times shorter than in macerate (Table 2). The rate of CA degradation is also well illustrated by the ln(C) graphs for extracts obtained in a water bath (B) (Figure 2A) and for extracts obtained using ultrasound (Figure 2B).

RA was very stable both in oil macerate and in ethane, methanol, and alcohol–water extracts (70%E, 70%M) stored for 28 days at refrigerated temperature (Figure 1C). In aqueous extracts, however, its degradation occurred in a way that most closely matched the second-order kinetic model (Figure 3, Table S2). The value of the rate constant k is about 10.2 [dm^3^/mol·d], while the time after which half of the RA content is degraded, i.e., the half-life (T_1/2_) is about 2.8 days (Table 2).

Degradation of active ingredients can be concentration-dependent. Generally, higher ingredient concentrations increase the reaction rate because the more “particles,” the more “collisions.” In a zero-order reaction, the reaction rate is constant and independent of the substrate concentration. Our studies have shown that carnosic acid degradation is a first-order reaction, meaning the rate of carnosic acid degradation is linearly dependent on its concentration. This relationship is also illustrated in the graphs (Figure 1A); the graph roughly resembles a straight line. In the case of rosemary acid degradation in water, the graph resembles a fragment of a parabola (Figure 1C), as the reaction rate depends quadratically on the concentration.

In summary: Sage oil macerates are rich in natural, highly stable ingredients. They can be used as a source of health-promoting bioactive ingredients for food enrichment, as well as antioxidant additives to inhibit oxidation. Due to the broad activity of Ca, C, and RA, such macerates can also be added to cosmetics and medical preparations. Our previous research [13] showed that such a concentrated macerate added to fish oil at 25% (*w*/*w*) can inhibit oxidation during refrigerated storage and at room temperature. Sage and oil are generally considered food products and are subject to general food law and food safety requirements. Generally, the macerate raises no objections, and there are no legal contraindications to its use in food. Furthermore, the natural components, the lack of organic solvents, and the low degree of processing make it a “green product” that can inspire consumer confidence. Only sage essential oil, which may leach into the macerate during extraction, may raise some safety concerns due to its α-thujone content, which requires further investigation in the near future.

## 3. Materials and Methods

### 3.1. Materials

Dried sage (*Salvia officinalis* L.), variety Bona, was obtained from a herb producer in Poland. Before the study the plant material was ground in a mill to a powder with a particle diameter of up to 0.4 mm and then stored in airtight plastic bags in a dark place until analysis, but no longer than four weeks. Fish oil—cod liver oil (LYSI, Reykjavik, Iceland) stabilized with tocopherol was purchased from a pharmacy in Szczecin. It was stored in a refrigerator at 4 °C. Analytically pure chemical reagents were used to prepare the extracts: methanol (Chempur, Piekary Śląskie, Poland), ethanol 99.8% (Avantor Performance Materials Poland S.A., Gliwice, Poland), and deionized water, as well as 70% methanol (*w*/*w*), and 70% ethanol (*w*/*w*), which were prepared the day before the study. HPLC solvents—glacial acetic acid, acetonitrile, water and high-purity (≥98%) standard substances (carnosol, carnosic acid, rosmarinic acid)—were from two manufacturers: Chempur, Piekary Śląskie, Poland, and Sigma-Aldrich, Darmstadt, Germany.

### 3.2. Oil Extraction—Preparation of Sage Macerates

Sage was mixed with fish oil in a glass bottle (mass ratio of sage to oil: 1 to 5.7) and placed in an ultrasonic bath (Ultron, Dywity, Poland; frequency 40 kHz, temperature 20–25 °C, power 320 W) for 60 min. Then, the macerate was filtered through a medium-soft qualitative filter on a Büchner funnel, using a vacuum pump.

Before each analysis, a portion of the macerate was taken to extract the active compounds from the macerate into the solvent. The macerate was dissolved in hexane and shaken with methanol or 70% methanol; then, the samples were extracted in an ultrasonic bath according to Hrebień-Filisińska & Tokarczyk (7). Two types of extracts were obtained from each macerate: methanol and methanol–water 70%, which were analyzed by HPLC as soon as possible. The macerate analyzed with methanol was marked: MA/O′, and the macerate analyzed with 70% methanol was marked: MA/O″.

### 3.3. Solvent Extraction—Preparation of Extracts

Ultrasonic extraction (U)

0.05 g of sage was weighed into glass bottles and 45 mL of solvent (methanol, ethanol, 70% ethanol, 70% methanol) was added. After closing, the bottles were placed in an ultrasonic bath for 10 min (temperature 20–25 °C). Then, the extracts were poured into measuring flasks (50 mL) and filled up to the mark with the appropriate solvent, then filtered through a filter paper into dark glass bottles.

Decoction—extraction in a “hot” water bath (B)

0.05 g of sage was weighed into round-bottomed flasks with a ground joint and 45 mL of solvent (methanol, ethanol, 70% ethanol, 70% methanol) was added. The sage components were extracted in a water bath with a reflux condenser for half an hour (temp. approx. 78 °C). Then, the samples were cooled, poured into 50 mL measuring flasks, and filled up to the mark with the appropriate solvent, then filtered through a filter paper.

Water infusions—infusion (I)

0.05 g of sage was weighed into glass bottles and approximately 45 mL of boiled hot deionized water was poured, covered, and left to cool, then poured into measuring flasks (50 mL) and filled with water to the mark and filtered through a filter paper.

All extracts were stored in a refrigerator at 4 °C for 28 days, while the oil macerate was stored for 15 weeks. At the beginning, samples for analysis were taken daily (for the first 16 days), then after 5 and 7 days, and the macerate was tested again after 6 and 15 weeks. The extracts obtained together with the markings are presented in Table 3.

### 3.4. HPLC Analyses

In all the extracts obtained, the following were determined: rosmarinic acid, carnosic acid, and carnosol. The identification of the active compounds was carried out by liquid chromatography (HPLC). An Agilent 1260 Infinity II liquid chromatograph coupled with a PDA detector was used. Separation was performed on a Nucleosil 120-5 C18 reversed-phase column, 250 × 4.6 mm, at room temperature. The mobile phase consisted of acetonitrile (solvent A) and water with 5% (*w*/*w*) acetic acid (solvent B). The flow was maintained at 0.5 mL/min. The gradient program was as follows: 15% A/85% B from 0 to 12 min, then changed linearly to 0% A/100% B after 30 min, then changed to 85% A and 15% B after 50 min, and the 85% A and 15% B system was maintained for another 10 min. The total analysis time was 60 min. The injection volume was 20 μL, and peaks were monitored at λ = 280 and 325 nm. Extracts were filtered through a 0.45 μm membrane filter before injection. Compounds were identified based on spectrum and retention time; retention time [minutes]: carnosic acid: 24.4–24.5; carnosol—22.6–22.8; rosmarinic acid—12.0. The concentration was determined in mg of a given compound per ml of extract, and then converted and presented in mg per g of sage or g of macerate. In the case of calculations related to the kinetic model of the reaction, the concentration was converted to mol/dm^3^ (assuming the molar mass value: 360.32 g/mol—for rosmarinic acid, 332.44 g/mol—for carnosic acid).

### 3.5. Kinetic Modeling

In the first stage, graphs of the dependence of the concentration of rosmarinic acid, carnosic acid, and carnosol on time were analyzed. The graphically presented data excluded that the reactions proceeded according to the zero-order model (there was no linear decrease in concentration); therefore, the first- and second-order kinetic models were taken into account for further analyses. In the case of carnosol, no model was fitted, because first an increase and then a decrease in concentration was observed during storage. Then, graphs of the functions for both models were drawn, according to Abedi et al. [25]; the function equations were determined and the fit coefficients (R^2^) for changes in the concentration of carnosolic and rosmarinic acid (for aqueous extracts) as a function of time were compared. For the sake of clarity, the base unit of time, second and derived units containing “second” (s^−1^; dm^3^/mol‧s), was converted to day [d]. For the first-order kinetic model, the reaction rate equation was expressed as follows:

dC/dt = −kC

ln(C/C_0_) = −kt

(where k—constant rate [d^−1^], C_0_—initial concentration [mol/dm^3^], C—concentration at a given moment [mol/dm^3^], t—time [d]).

In the case of the second-order kinetics model, the reaction rate was presented as follows:

dC/dt = −kC^2^

1/C = 1/C_0_ + kt

Then, after determining the kinetic model, the rate constant k was determined graphically (based on the equation of the function, in which the slope of the line to the X-axis is determined) and algebraically, using the following formulas:

k = ln(C/C_0_) [d^−1^]—for first-order models

k = ln((C^−1^ − C_0_^−1^)/t) [dm^3^/mol·d]—for second-order models

(where C_0_—initial concentration [mol/dm^3^], C—concentration at a given moment [mol/dm^3^], t—time [d]).

The half-life time T_1/2_ was also determined, substituting the value determined graphically and algebraically for k. The following formulas were used:

T_1/2_ = ln(2)/k—for first-order models,

T_1/2_ = 1/(kC_0_)—for second-order models.

### 3.6. Statistical Analysis

Tukey’s test for *p* < 0.05 was used to assess the significance of differences. The results were statistically processed using the STATISTICA program (version 12.3).

## 4. Conclusions

Ultrasound-assisted maceration produces a macerate rich in carnosic acid, with high storage stability. Edible oil can be used for extraction as a green extractant instead of organic solvents to isolate carnosic acid from sage. Although the oil extracts slightly smaller amounts of this compound than ethanol or methanol, the advantage of using oil is that the carnosic acid in it is very stable. The level of carnosic acid in methanol extracts after about 3 days of storage at refrigeration temperature equalized with the level in oil, and in ethanol extracts, after 28 days, the CA concentration dropped below that for oil. CA degradation proceeded according to the first-order kinetic model, with very little noticeable changes in oil, hence a fairly low-fitting coefficient R^2^ for this model was obtained. The constant rate of CA degradation reaction in oil was approximately 10 times lower than in ethanol extracts, and as much as 153–169 times lower than in methanol–water extracts (70%). In most cases, alcohol extracts obtained by high-temperature solvent leaching (in a water bath), compared to “cold” extracts after ultrasound, were less stable for CA. However, the greatest impact on both extraction efficiency and CA stability was exerted by the type of solvent used. It was noted that the more polar the extractant, the faster the CA degradation occurred in it. The C concentration determined in the macerate after extraction was one of the lowest, compared to the other extracts, because its presence is most likely closely related to the CA degradation process. On the other hand, oil is not suitable for RA extraction. The best solvent for RA was 70% ethanol and methanol, followed by water and concentrated alcohols. In all extracts, except water, RA was very stable. In water, the kinetics of its degradation reaction corresponded most closely to the second-order model. When preparing aqueous extracts, e.g., in the form of sage tea, one should bear in mind that they can be quite a good source of rosmarinic acid, provided that they are drunk immediately after preparation.

## Figures and Tables

**Table 1:** Content of carnosic acid (CA), carnosol (C) and rosmarinic acid (RA) in oil macerate (MA/O) and in various extracts, calculated converted into sage mass or macerate mass * [mg/g].

Extraction/Extractant	CA	C	RA
I/W	n/i	n/i	12.45 ± 0.27 ^a^
B/70M	6.92 ± 0.89 ^b^	4.01 ± 0.35 ^a^	19.87 ± 0.52 ^b^
B/M	9.40 ± 0.41 ^a^	2.20 ± 0.06 ^b^	11.50 ± 1.01 ^a^
B/70E	6.96 ± 0.76 ^b^	3.64 ± 0.04 ^a^	19.16 ± 1.21 ^b^
B/E	9.90 ± 0.63 ^a^	n/i	7.56 ± 0.61 ^d^
U/70M	7.53 ± 1.04 ^b^	3.08 ± 0.61 ^a^	19.46 ± 0.91 ^b^
U/M	9.30 ± 0.67 ^a^	2.31 ± 0.05 ^b^	8.10 ± 0.08 ^d^
U/70E	7.54 ± 0.94 ^b^	3.68 ± 0.06 ^a^	20.24 ± 1.01 ^b^
U/E	10.10 ± 0.42 ^a^	n/i	3.86 ± 0.21 ^e^
MA/O′	7.10 ± 0.12 ^b^	1.03 ± 0.11 ^c^	n/i
MA/O′	1.24 ± 0.02 *	0.18 ± 0.02 *	0.11 ± 0.001 ^c^
MA/O″	5.91 ± 0.27 ^c^	2.15 ± 0.05 ^b^	0.02 ± 0.001 *
MA/O″	1.03 ± 0.05 *	0.38 ± 0.01 *	12.45 ± 0.27 ^a^

W—water, 70M—70% methanol, M—methanol, 70E—70% ethanol, E—ethanol, O—oil; extraction method: U—in an ultrasonic bath, B—in a water bath, I—infusions, MA—maceration; ′ methanol was used for the analyses; ″ 70% methanol was used for the analyses; * mg/g macerate; n/i—not identified; ^a^, ^b^, ^c^, ^d^, ^e^—values marked with a different lowercase letter differ significantly (*p* < 0.05).

**Table 2:** Rate constant k and half-life of carnosic acid and rosmarinic acid in macerate and various extracts.

No.	Sample	Carnosic Acid (CA)—First-Order Kinetic			
		Constant Rate		Half-Life T_1/2_ [d]	
		Algebraic k [d^−1^]	Graphic	Algebraic	Graphic
1	MA/O′	0.00188	0.0024	369.23	288.8
2	U/E	0.01884	0.016	36.79	43.32
3	B/E	0.0198	0.0189	35.07	36.67
4	U/M	0.10264	0.1065	6.75	6.51
5	B/M	0.1190	0.1257	5.82	5.51
6	U/70E	0.21979	0.2443	3.15	2.84
7	B/70E	0.2512	0.2619	2.76	2.65
8	B/70M	0.2867	0.348	2.42	1.99
9	U/70M	0.31756	0.3854	2.18	1.80
		Rosmarinic acid (RA)—Second-order kinetic			
	I/W	10.18839 *	11.901	2.84	2.43

W—water, 70M—70% methanol, M—methanol, 70E—70% ethanol, E—ethanol, O—oil; extraction method: U—in an ultrasonic bath, B—in a water bath, I—infusions, MA—maceration; ′ methanol was used for the analyses; * [dm^3^/mol·d].

**Table 3:** Sage extraction methods, solvents/extractant, and extract markings used in the studies.

Extraction Method	Solvent/Extractant	Marking Sample
Infusions	Water	I/W
Water bath/hot	70% methanol	B/70M
	Methanol	B/M
	70% ethanol	B/70E
	Ethanol	B/E
Ultrasonic washer	70% methanol	U/70M
	Methanol	U/M
	70% ethanol	U/70E
	Ethanol	U/E
Oil maceration	Oil	MA/O′
	Oil	MA/O″

′ methanol was used for the analyses ″ 70% methanol was used for the analyses.

## Data Availability

The data used to support the findings of this study are available from the corresponding author upon request.

## Supplementary Materials

The following supporting information can be downloaded at: https://www.mdpi.com/article/10.3390/molecules30224394/s1, Table S1: Retention of carnosic acid (CA), carnosol (C) and rosmarinic acid (RA) in sage oil macerate during 15 weeks of storage at refrigerated temperature; Table S2: Equations of kinetic models of carnosic and rosmarinic acid degradation.
