# Supplementary material for: Assessment of Stability and Degradation Kinetics of Carnosic and Rosmarinic Acid in Edible Oil and Its Effectiveness as an Extraction Medium Compared to Other Solvents
Source: Molecules. 2025 Nov 13;30(22):4394. doi: 10.3390/molecules30224394 (PMC12655447; doi:10.3390/molecules30224394)
Supplement: Supplementary file 1 [file molecules-30-04394-s001.zip › molecules-3938712-supplementary.pdf]

Supplementary Materials

Table S1. Retention of carnosic acid (CA), carnosol (C) and rosmarinic acid (RA) in sage oil macerate during 15 weeks of storage at refrigerated temperature (MA/O' – methanol extract of macerate; MA/O'' – methanol-water 70% extract of macerate; n/i -not identified).

Parameter /sample	Day 1	6 weeks	15 weeks
CA [mg/g]			
MA/O'	7.10 ±0.35 [a]	7.07 ±0.42 [a]	6.49 ±0.12 [b]
MA/O''	5.91 ±0.25 [a]	5.92 ±0.23 [a]	5.98 ±0.15 [a]
C [mg/g]			
MA/O'	1.03 ±0.05 [a]	0.95 ±0.08 [a]	1.00 ±0.09 [a]
MA/O''	2.15 ±0.11 [a]	1.25 ±0.25 [b]	1.15 ±0.18 [b]
RA [mg/g]			
MA/O'	n/i	n/i	n/i
MA/O''	0.11 ±0.02 [a]	0.11 ±0.03 [a]	0.12 ±0.05 [a]

a, b - values marked with a different lowercase letter differ significantly ($p < 0.05$).

Table S2. Equations of kinetic models of carnosic and rosmarinic acid degradation.

Sample	First-order kinetic y=lnC		Second-order kinetic y=1/C	
	Carnosic acid (CA)			
B/70M	y = -0.348x – 3.56	R^2 = 0.9929	y = 0.1116x + 0.0032	R^2 = 0.9538
B/M	y = -0.1257x – 3.426	R^2 = 0.9845	y = 0.0366x + 0.0056	R^2 = 0.9388
B/70E	y = -0.2619x – 3.714	R^2 = 0.9734	y = 0.101x - 0.0002	R^2 = 0.8997
B/E	y = -0.0189x – 3.455	R^2 = 0.9503	y = 0.0003x + 0.14	R^2 = 0.6375
U/70M	y = -0.3854x – 3.479	R^2 = 0.9888	y = 0.1259x - 0.0205	R^2 = 0.9698
U/M	y = -0.1065x – 3.459	R^2 = 0.9825	y = 0.027x + 0.0372	R^2 = 0.9405
U/70E	y = -0.2443x – 3.464	R^2 = 0.9743	y = 0.0834x - 0.0344	R^2 = 0.8547
U/E	y = -0016x – 3.4318	R^2 = 0.8487	y = 0.002x + 0.0901	R^2 = 0.7853
**MA/O'**	**y = -0.0024x - 5.499**	**R^2 = 0.6311**	**y = 0.0003x + 0.14**	**R^2 = 0.6375**
	Rosmarinic acid (RA)			
I/W	y = -0.0885x - 3.978	R^2 = 0.7603	y = 11.901x + 38.181	R^2 = 0.9235

W – water, 70M -70% methanol, M - methanol, 70E - 70% ethanol, E - ethanol, O – oil; extraction method: U - in an ultrasonic bath, B - in a water bath, I - infusions, MA - maceration; ' methanol was used for the analyses.
